# Braking Time and Cognitive, Visual, and Motor Determinants in Older Drivers With Type 2 Diabetes Mellitus With and Without Diabetic Peripheral Neuropathy: A Controlled Cross‐Sectional Study

**DOI:** 10.1111/1753-0407.70269

**Published:** 2026-10-04

**Authors:** Vivian Rodrigues de Matos Souza, Matheus Henrique dos Santos Lino, Julia Maria D'Andréa Greve, Guilherme Carlos Brech, Vanderlei Carneiro da Silva, Angelica Castilho Alonso

**Affiliations:** ^1^ Graduate Program in Aging Sciences at University São Judas Tadeu São Paulo Brazil; ^2^ Researcher at the Movement Study Laboratory of the Hospital das Clínicas at the School of Medicine of the University of São Paulo São Paulo Brazil

**Keywords:** aged, automobiles, diabetic neuropathies, Type 2 diabetes mellitus

## Abstract

**Background:**

Driving is a complex task that requires efficient integration of sensorimotor, visual, and cognitive systems. Although diabetic peripheral neuropathy (DPN) is associated with reduced driving performance, the impact of this condition when combined with aging is still poorly understood.

**Aims:**

To assess braking time in older drivers with Type 2 diabetes mellitus (T2DM), with and without DPN, comparing them with a control group and investigating associations with motor, cognitive, and visual characteristics.

**Methods:**

Cross‐sectional study involving individuals aged 60–79 years, distributed into three groups: T2DM without DPN (*n* = 17), T2DM with DPN (*n* = 22), and control (*n* = 25). DPN was identified using the Michigan Neuropathy Screening Instrument, Brazilian version (MNSI‐Brazil), with a cutoff score ≥ 2.5. Cognitive (Montreal Cognitive Assessment—MoCA, cognitive TUG), visual (acuity, stereopsis, color vision, and adaptometry), and motor assessments (handgrip strength and plantar flexor strength) were performed, along with a driving simulation.

**Results:**

Older adults with T2DM showed longer braking times than the control group (*p* < 0.01), regardless of the presence of DPN. Participants with DPN presented lower upper‐limb strength (*p* = 0.01) and reduced strength of the right plantar flexor muscles (*p* < 0.01). Braking time was influenced by sex and plantar strength.

**Conclusion:**

T2DM is associated with increased braking time in older adults, and reduced plantar flexor muscle strength, particularly among women, highlights the need for monitoring and targeted muscle strengthening to preserve driving safety and autonomy.

## Highlights

Older drivers with T2DM show significantly longer braking times compared to those without diabetes. Female sex, older age, and reduced plantar flexor strength predict poorer braking performance. These findings highlight how metabolic, physiological, and sex‐related factors influence response capacity in situations requiring motor agility.

## Graphical abstract

Older drivers with T2DM show significantly longer braking times compared to those without diabetes, regardless of the presence of peripheral neuropathy based on the Michigan Neuropathy Screening Instrument. Female sex and reduced plantar flexor strength predict poorer braking performance. These findings highlight how metabolic, physiological, and sex‐related factors influence response capacity in situations requiring motor agility.

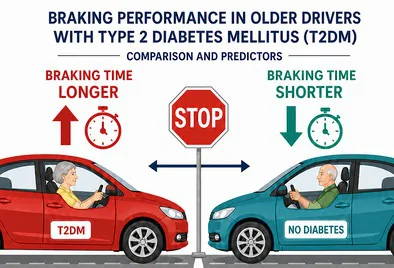

## 1 Introduction

Aging is a natural process [1, 2], often associated with reduced functional capacity [3] and an increased incidence of chronic diseases [4, 5], which can directly impact mobility and safety in performing daily activities such as driving [6].

Among the most prevalent chronic conditions in the older population is Type 2 diabetes mellitus (T2DM), a systemic and multifactorial disease characterized by persistent hyperglycemia resulting from insulin resistance and/or impaired insulin secretion [4, 5], affecting approximately 10.5% of the Brazilian population aged 20 to 79 years [7].

Among the most frequent complications of T2DM, diabetic peripheral neuropathy (DPN) stands out [8]. It is a progressive dysfunction of the peripheral nervous system resulting from chronic hyperglycemia, affecting sensory and motor fibers, especially in the lower limbs. This sensorimotor dysfunction can lead to muscle atrophy and weakness, as well as deficits in sensation and proprioception [9, 10, 11, 12], resulting in reduced ankle range of motion and functional alterations in the lower limbs, with potential impact on driving performance [13, 14].

Driving is an activity that promotes autonomy, social participation, and well‐being [15]. However, the decline in cognitive, sensory, and motor functions associated with aging may compromise driving performance and increase the risk of accidents [16, 17, 18, 19, 20]. Moreover, the sensorimotor deficits resulting from DPN directly affect tasks that require motor precision, such as pedal control [21, 22].

Driving is a complex task that depends on the efficient integration of sensorimotor, visual, and cognitive systems [16]. Braking, in particular, requires rapid and coordinated responses involving perception, information processing, and motor execution [17, 18]. Braking time, defined as the interval between the perception of a stimulus and the activation of the brake, is an important indicator of a driver's response capacity [23, 24, 25].

Meyr and Spiess [13] observed that drivers with T2DM and DPN exhibited significantly slower braking times compared with the control group. In a subsequent study, Spiess et al. [14] found that individuals with T2DM and DPN had longer braking times than those with T2DM without DPN, while the average brake response time among participants without DPN remained below the established safety threshold (0.70 s). In turn, Sansosti et al. [26] reported that foot complications—such as lesions, amputations, or Charcot arthropathy—did not significantly worsen braking time, suggesting that diabetes alone already impairs motor response. However, these studies did not specifically include older adults, limiting the understanding of the combined effects of aging and diabetes on driving performance.

Considering the substantial increase in the number of older drivers and the high prevalence of T2DM in this age group, understanding the impact of these conditions on braking time is essential for traffic safety and for maintaining functional autonomy. Moreover, the absence of studies evaluating older drivers with T2DM and DPN highlights an important scientific gap. In this context, the present study aims to assess braking time in older drivers with T2DM, with and without DPN, comparing them with a control group and investigating associations with motor, cognitive, and visual characteristics.

Recent evidence from naturalistic driving studies has reinforced the association between DM and impaired driving safety in older adults. Liu et al. [27] in the LongROAD Study demonstrated that older drivers with DM, particularly insulin users, exhibited a higher incidence of hard braking events during real‐world driving. Although naturalistic driving studies have demonstrated increased driving risk among older adults with DM, particularly insulin users, the neuromuscular and sensorimotor mechanisms underlying impaired braking performance remain poorly understood. In this context, investigating braking time and its association with DPN and lower‐limb muscle strength may contribute to understanding functional factors associated with driving safety.

## 2 Methods

### 2.1 Study Design and Ethics

This is a controlled, cross‐sectional, quantitative study approved by the Ethics Committee of Universidade São Judas Tadeu, CAAE No. 83378424.2.0000.0089. All participants provided written informed consent before taking part in any procedures in the present study.

### 2.2 Study Setting

The study was conducted at Universidade São Judas Tadeu (USJT) in partnership with the Movement Study Laboratory (LEM) of the Institute of Orthopedics and Traumatology at the Hospital das Clínicas, Faculdade de Medicina, Universidade de São Paulo (IOT‐HC‐FMUSP).

### 2.3 Participants and Recruitment

Participants with T2DM were recruited through convenience sampling from outpatient clinics and community advertisements distributed via social media.

A total of 72 older drivers were evaluated and divided into three groups: (a) 17 with T2DM without DPN (SNPD—11 men and 6 women); (b) 22 with T2DM with DPN (DPN—17 men and 5 women); (c) 25 healthy older drivers (Control—13 men and 12 women), recruited through social media and WhatsApp groups.

The inclusion criteria were: having no major limitations in joint movement of the ankles, knees, hips, or cervical spine, whether due to a history of injury or surgery that could compromise the ability to operate a motor vehicle; not using medications that could impair driving fitness, such as benzodiazepines, sedatives, antipsychotics, and centrally acting medications, were excluded from participation and achieving a score above 26 points on the MoCA for individuals with at least 8 years of schooling. For the T2DM groups, an additional requirement was a diagnosis of T2DM, with or without DPN, for more than 5 years.

The exclusion criterion was older drivers who, for any reason, were unable to perform the tests used in the assessment.

### 2.4 Procedures

In a single session at the LEM, all participants completed a questionnaire related to personal information, sociodemographic data, and driving history, and underwent functional tests administered by the researcher, who is a physiotherapist experienced in these assessments. The session lasted approximately one and a half hours.

### 2.5 Assessments

#### 2.5.1 Classification of Diabetic Peripheral Neuropathy

##### 2.5.1.1 Michigan Neuropathy Screening Instrument (MNSI–Brazil)

The instrument consists of two stages. The first is a self‐reported questionnaire on signs and symptoms in the lower limbs, composed of 15 questions addressing aspects such as pain, tingling, numbness, cramps, and sensory changes in the feet and legs, with “yes” or “no” answers for each item. The second stage corresponds to the physical examination, which includes: inspection of the feet to check for calluses, fissures, lesions, infections, deformities, and amputations; assessment of tactile sensitivity using a 10 g monofilament (Semmes–Weinstein) applied to three specific points on the foot; and evaluation of vibratory sensitivity using a 128 Hz tuning fork placed on the hallux [28]. To classify participants with DPN, a fuzzy logic–based classification system was used [29]. The “neuropathy grade score” applied was: DPN absent (< 2.5) and DPN present (≥ 2.5) [30].

#### 2.5.2 Cognitive Assessments

##### 2.5.2.1 Montreal Cognitive Assessment (MoCA)

This is a screening tool for mild cognitive impairment that evaluates cognitive domains such as attention and concentration, executive functions, memory, language, visuoconstructive skills, abstraction, calculation, and orientation. It takes approximately 10 min to administer. The total score is 30 points, with scores of 26 or higher considered normal for individuals with at least 8 years of schooling [31].

##### 2.5.2.2 Timed Up and Go Test With Cognitive Task (TUG + COG)

This is a variation of the traditional TUG test that, in addition to assessing physical mobility and motor performance, also evaluates the ability to perform simultaneous tasks (dual‐tasking) by combining motor and cognitive activities. In the TUG + COG procedure, the participant performs the same TUG test while completing an additional cognitive task across three different trials: naming colors, animals, and fruits. The execution time for each trial is recorded in seconds, and the average of these times represents the final result. The classification of times follows the same criteria as the original TUG test.

#### 2.5.3 Visual Assessment

The visual screening was performed using the Raizamed 2000 device. Visual acuity was measured using the Snellen optometric chart, in which normal vision is 20/20, and the score was obtained according to the number of correctly identified letters (0.5 points per letter). Stereopsis was assessed through binocular depth perception. Color vision was analyzed by correctly identifying the colors green, yellow, and red, while adaptometry (glare recovery) evaluated the ability to recognize letters after exposure to intense light.

#### 2.5.4 Motor and Physical Performance Assessments

##### 2.5.4.1 Assessment of Plantar Flexor Muscle Strength

Strength was evaluated using an isokinetic dynamometer (Biodex System 2). Participants were positioned seated, with support under the thigh and foot, the knee at 30° of flexion, and secured with belts and Velcro straps. They performed three submaximal attempts for familiarization, followed by two test sets of five repetitions at 30°/s, starting with the dominant limb (only the second test was used for analysis) [32]. The peak torque relative to body weight (PT/BW, %) of the five repetitions of the plantar flexor muscles was assessed.

##### 2.5.4.2 Measurement of Handgrip Strength

A Jamar dynamometer was used, with the participant seated, arm parallel to the body, shoulder adducted, elbow flexed at 90°, and forearm and wrist in a neutral position. Three strength measurements were taken for both dominant and nondominant hands, with a 1‐min interval between attempts. For analysis, the arithmetic mean of the three values obtained was considered, expressed in kilogram‐force (kgf) [33].

##### 2.5.4.3 Measurement of Dynamic Balance and Functional Mobility

The Timed Up and Go Test (TUG) was used, a clinical functional mobility test that assesses balance, gait speed, and functional capacity. The time, in seconds, required for the individual to rise from a standardized chair, walk three meters, return, and sit down was recorded [34]. Participants also completed the TUG with a dual task (TUG + COG), naming colors, animals, and fruits, with the final result based on the average time across the three trials [16, 34].

##### 2.5.4.4 Driving Simulator Test

The test was performed using a “Car‐Simulator Trainer—Type F12PT” (FoerstGMBh), similar to a vehicle equipped with a steering wheel, dashboard with speedometer, brake, accelerator, and clutch pedals, gear shift, seat, seatbelt, and headlights. The driving route was displayed on three 42″ LCD monitor screens. Braking time was measured by the device as reaction time. Participants were instructed to sit in the driving simulator so the seat, seatbelt, and mirrors could be adjusted. The test began with a familiarization drive on a 3.3 km traffic‐free highway scenario. After this circuit, the actual test was started on the same type of road. During the route, the word “stop” appeared randomly, and the driver had to bring the car to a complete stop. This command was repeated five times. The parameters evaluated were: braking time, calculated as the arithmetic mean of the five trials, and average speed [16].

### 2.6 Data Analysis Procedures

The data were presented as mean and standard deviation. Initially, the clinical and functional characteristics of the participants, including glycated hemoglobin, time since diagnosis, age, and the results of the visual, cognitive, and motor tests, were compared among the three groups (control, T2DM with and without DPN) using analysis of variance (ANOVA). For each variable, the *p*‐value, effect size (*η* ^2^ *p*), and Bonferroni post hoc comparisons were reported when applicable.

A multiple linear regression model was developed to assess the association between the presence of T2DM and DPN (compared to the control group) and braking time. The model was adjusted for age, sex, and the ratio between body weight and plantar flexor peak torque (BW/PT). Age and sex were included as covariates due to their potential influence on motor performance, cognitive processing, and braking response in older adults. The assumptions of ANOVA and linear regression were verified: normality, homogeneity of variances, linearity, homoscedasticity, and absence of multicollinearity (assessed by VIF). The significance level adopted was 5% (*p* < 0.05), and the analyses were performed using the Jamovi software (version 2.4.14.0).

## 3 Results

Regarding braking time, participants with T2DM, regardless of whether they had DPN, took longer to brake the car compared to the control group (*p* < 0.01). The results show that older adults with DPN exhibited lower muscle strength compared to the control group, both in the upper limbs (handgrip strength on the dominant side, *p* = 0.01) and in the plantar flexor muscles of the right side (*p* < 0.01). The characterization of participants in terms of age, disease status, visual tests, motor tests, and cognitive screening is described in Table 1.

**TABLE 1:** Comparison of clinical and functional characteristics and braking time among older drivers with and without diabetic peripheral neuropathy and the control group.

	Without DPN	DPN	CONTROL	*p*	*η* ^2^ *p*	Post hoc Bonferroni
Age (years)	70.2 (4.7)	71.5 (4.4)	71.4 (5.2)	0.61	0.17	
Glycated hemoglobin (%)	7.1 (1.5)	7.2 (1.0)	—	0.84	0.003	
Time since diagnosis (years)	18.1 (10.5)	23.4 (15.1)	—	0.44	0.04	
Neuropathy grade	1.5 (0.6)	5.9 (2.1)	—	*p* < 0.01*	0.70	
Visual tests						
Snellen R	5.4 (3.0)	4.9 (3.1)	4.2 (2.9)	0.35	0.03	
Snellen L	5.4 (3.5)	4.8 (3.2)	2.7 (1.9)	0.41	0.01	
Binocular	2.0 (1.8)	1.8 (1.5)	1.1 (1.4)	0.64	0.00	
Cognitive tests						
MoCA	25.5 (2.1)	25.4 (2.7)	24.2 (3.4)	0.24	0.04	
Cognitive TUG (s)	9.5 (1.8)	10.4 (3.4)	10.0 (2.8)	0.43	0.03	
Motor tests						
TUG (s)	8.2 (1.5)	8.8 (2.6)	8.3 (1.6)	0.37	0.03	
Dominant handgrip strength (kgf)	31.4 (7.7)	31.2 (9.1)^a^	34.7 (10.7)^a^	0.01*	0.15	*a* = 0.01
Non‐dominant handgrip strength (kgf)	30.2 (8.9)	29.5 (7.9)	31.4 (9.8)	0.14	0.06	
BW/PT right plantar flexor (%)	76.8 (29.9)	56.4 (19.1)^a^	84.6 (21.3)^a^	*p* < 0.01*	0.24	*a* = *p* < 0.01
Simulator test						
Braking time (s)	0.97 (0.17)^b^	1.00 (0.18)^a^	0.86 (0.11)^a^, ^b^	*p* < 0.01*	0.24	*a* = *p* < 0.01 *b* = *p* < 0.01
Speed (km/h)	54.8 (12.0)	54.9 (13.8)	57.11 (9.3)	0.570	0.02	

*Note:* General linear Model *p < 0.05.

Abbreviations: BW/PT—body weight/peak torque ratio; DPN—diabetic peripheral neuropathy; L—left; MoCA—Montreal cognitive assessment; R—right; TUG—timed up and go.

^a^ Difference between individuals with DPN compared to the control group.

^b^ Difference between individuals without DPN compared to the control group.

In Table 2, the linear regression model for braking time showed a good fit (adjusted *R* ^2^ = 0.454). After controlling for sex, age, and the body weight/peak torque ratio (BW/PT), it was observed that older adults with T2DM, both without DPN and with DPN, had longer braking times compared to the control group (*β* = 0.14; *p* = 0.005 and *β* = 0.12; *p* = 0.026, respectively).

**TABLE 2:** Model fit measures for braking time according to neuropathy, sex, and age.

Predictor	Estimates	Standard error	*t*	*p*	Adjusted *R* ^2^
Intercept^a^	0.4610	0.3176	1.45	0.155	0.454
Group					
Without DPN—control	0.1445	0.0487	2.96	*p* < 0.01*	
DPN—control	0.1179	0.0507	2.33	0.026*	
Sex					
Female—Male	0.1308	0.0393	3.32	*p* < 0.01*	
Age	0.0069	0.0039	1.76	0.087	
BW/PT of plantar flexors (%)	−0.0018	0.0009	−2.14	0.039*	

Abbreviations: BW/PT—body weight/peak torque ratio; DPN—diabetic peripheral neuropathy. *p < 0.05.

^a^ Represents the reference level.

Sex was also a significant predictor, indicating that women had longer braking times than men (*β* = 0.13; *p* = 0.002). On the other hand, the BW/PT ratio was negatively associated with braking time (*β* = −0.0018; *p* = 0.039), suggesting that the lower the muscle strength of the plantar flexors, the longer the time required to brake the car.

## 4 Discussion

The main findings of this study showed that older drivers with T2DM had significantly longer braking times compared to individuals without T2DM, regardless of the presence of DPN. These findings suggest that participants with T2DM, even in the absence of clinical DPN, may experience impairments in the sensory and motor performance required for safe driving. It was also observed that the presence of T2DM, female sex, and lower plantar flexor muscle strength were predictors of poorer braking performance, indicating that metabolic and physiological factors influence motor responses.

The increased braking time observed in participants with T2DM is consistent with previous findings, although earlier studies were not conducted exclusively with older adults. Recent prospective evidence from the LongROAD Study further demonstrated a higher incidence of hard braking events among older drivers with diabetes, particularly insulin users, reinforcing the association between diabetes and impaired driving safety in real‐world conditions. Meyr and Spiess [13] identified braking times that were 37.9% slower in diabetics with DPN compared to controls, while Spiess, Sansosti, and Meyr [14] demonstrated that DPN has a negative effect on braking time (11.49% slower) when compared to individuals with diabetes without DPN. Complementarily, Sansosti, Spiess, and Meyr [26] found that foot complications caused by DPN did not further worsen performance, indicating that the determining factor is T2DM and its diffuse neuromotor alterations. Thus, the findings of the present study expand this evidence by showing that, in the context of aging, T2DM further compromises neural processing speed, strength, and motor coordination, resulting in longer braking times and a potential negative impact on driving safety.

Although the variable age showed a *p*‐value of 0.08 in the overall model, it remained associated with braking time when adjusted for sex and muscle strength, explaining 45% of the outcome. Alonso et al. [16] demonstrated that older drivers exhibit reaction times 17% longer than middle‐aged adults, attributed to reduced muscle strength, postural balance, and cognitive functions. Similarly, Canonica et al. [35] identified age, muscle strength, and balance as significant predictors of braking time in older adults, while Silva et al. [36] linked aging to an increased risk of collisions. De Biase et al. [37] also confirmed that age, combined with motor, visual, and cognitive variables, explains a large portion of the variability in braking time.

The poorer braking performance observed among women appears to result from a multifactorial combination of neurophysiological, physiological, and sociocultural factors. Although no studies to date have focused specifically on individuals with T2DM, findings are available for older adult populations in general. From a neurophysiological standpoint, there is a component primarily related to hormonal modulation (estrogen) and the functional organization of cortical and subcortical areas involved in attention, executive control, and processing speed, such as the anterior cingulate cortex, prefrontal cortex, and basal ganglia. This modulation may influence the ability to shift attentional focus and inhibit competing stimuli, which affects performance in tasks requiring rapid responses under multiple demands, such as braking [38, 39, 40]. Casamento‐Moran et al. [41] demonstrated that although overall braking time is similar between sexes, the cognitive and motor components differ significantly, with cognitive processing being 27.4% slower in women, while motor execution is 24.3% slower in men. These differences suggest distinct patterns of neural recruitment between men and women, characterizing a phenomenon rooted in neurophysiology [41, 42]. Physiologically, the lower muscle strength and reduced motor integration often observed in women, especially older women, may negatively affect efficient braking time [36]. Finally, sociocultural factors, such as the fact that women, on average, drive less frequently and are less exposed to high‐risk traffic situations, may further contribute to longer response times [16, 37].

The results of the present study showed that participants with T2DM and DPN had lower plantar flexor muscle strength compared to controls. The decrease in muscle strength in these individuals is related to chronic hyperglycemia, which promotes protein glycation, oxidative stress and inflammation, as well as degeneration of motor and sensory nerve fibers characteristic of DPN, leading to neurogenic atrophy and proprioceptive deficits in the lower limbs [30, 43, 44, 45]. Furthermore, reduced glucose tolerance impairs distal lower‐limb muscle strength (plantar flexors) due to the increase in noncontractile intramuscular tissue (adipose tissue) [46]. This neuromuscular impairment, combined with aging, reduces the ability to generate force rapidly, affecting activities that require quick motor responses, such as the precise manipulation of the accelerator and brake pedals, resulting in longer braking time [3, 13, 47]. Interestingly, although braking time results for the groups with or without DPN were not significantly different from each other in Tables 1 and 2, the greater attenuation of the braking time difference for the DPN group after adjustment for plantar flexor strength in Table 2 suggests that lower‐limb muscle weakness may partially mediate the relationship between DPN and braking performance. This finding should be interpreted cautiously due to the limited sample size, but it may indicate a potential mechanistic pathway linking neuromuscular impairment and delayed braking responses in older adults with T2DM.

Handgrip strength, recognized as a central marker of sarcopenia [48] and a global indicator of overall muscle strength [49], is also considered a fitness parameter for driving according to the Brazilian National Traffic Council (CONTRAN, Resolution No. 425/2012), which establishes a minimum strength of 20 kgf in each hand for categories A and B. Although individuals with T2DM and, especially, those with DPN may present significant reduction in handgrip strength due to loss of motor units and sensorimotor alterations, the linear regression analysis of the present study did not show an association between this variable and braking time. This result suggests that upper‐limb weakness, although present in individuals with T2DM and DPN, does not directly influence the execution of this specific task, which depends predominantly on lower‐limb motor response. Similarly, functional assessment using the TUG test, a marker of mobility and physical performance [49, 50], did not show statistically significant differences between groups, although the studies by Alonso et al. [16] and De Biase et al. [37] in older adults without T2DM indicate that poorer performance on this test is associated with longer braking time. Thus, the findings reinforce that, in older adults with T2DM and DPN, the muscular integrity of the lower limbs is the most decisive component for safety and efficiency during driving.

### 4.1 Limitations, Clinical Implications, and Future Directions

Some limitations should be considered when interpreting the results. Although driving simulators cannot fully reproduce the sensory, motor, and emotional demands of real traffic environments, they provide a safe and standardized setting for investigating motor and cognitive responses associated with braking performance. Recent naturalistic driving studies have also demonstrated that hard braking events are strongly associated with driving safety outcomes in older adults, supporting the clinical relevance of braking performance assessment. The cross‐sectional design prevents establishing causal relationships between muscle strength, DPN, and driving performance. Moreover, the use of convenience sampling and recruitment through community advertisements and social media may have introduced selection bias, limiting the generalizability of the findings to the broader older adult population with T2DM. Differences in sex distribution across groups may also have influenced the results, despite adjustment for sex in the regression analyses. In addition, nutritional status, physical activity levels, and formal sarcopenia assessment were not comprehensively evaluated and may have influenced muscle performance and braking responses. Finally, the absence of direct neurophysiological measures, such as peripheral nerve conduction or functional neuroimaging, limits a more comprehensive understanding of the central and peripheral mechanisms underlying prolonged braking time.

Clinically, the findings indicate that older adults with T2DM, with and without DPN, exhibit slower motor responses during driving, such as braking, which increases the likelihood of accidents. Assessing lower‐limb muscle strength may help identify patients with functional impairments and guide specific interventions, such as resistance exercises and sensorimotor training, considering sex as a determining variable to optimize safety and independence in activities that require rapid responses.

Future longitudinal studies with larger and more representative samples should investigate the combined influence of neuromuscular, nutritional, and behavioral factors on driving performance in older adults with T2DM.

## 5 Conclusion

The results of this study indicate that older drivers with T2DM have a significantly longer braking time compared to individuals without diabetes, regardless of the presence of DPN. In addition, female sex and lower plantar flexor muscle strength were identified as predictors of poorer braking performance, demonstrating that metabolic, physiological, and sex‐related factors directly influence response capacity in situations that require motor agility.

## Author Contributions

This investigation was made possible by the efforts of a multidisciplinary and international team. **Vivian Rodrigues de Matos Souza**, **Angelica Castilho Alonso**, and **Julia Maria D'Andréa Greve:** conceptualization. **Vivian Rodrigues de Matos Souza**, **Matheus Henrique dos Santos Lino**, and **Angelica Castilho Alonso:** methodology. **Angelica Castilho Alonso** and **Vanderlei Carneiro da Silva:** formal analysis. **Vanderlei Carneiro da Silva** and **Angelica Castilho Alonso:** data curation/analysis. **Guilherme Carlos Brech**, **Matheus Henrique dos Santos Lino**, **Vivian Rodrigues de Matos Souza**, and **Angelica Castilho Alonso:** writing – original draft preparation. **Angelica Castilho Alonso**, **Julia Maria D'Andréa Greve**, and **Guilherme Carlos Brech:** writing – review and editing. **Angelica Castilho Alonso:** supervision. **Angelica Castilho Alonso** and **Guilherme Carlos Brech:** funding acquisition. All authors have read and agreed to the published version of the manuscript.

## Funding

This study was financed in part by the Coordenação de Aperfeiçoamento de Pessoal de Nível Superior—Brasil (CAPES)—Finance Code 001.

## Conflicts of Interest

The authors declare no conflicts of interest.

## Data Availability

The data that support the findings of this study are available on request from the corresponding author. The data are not publicly available due to privacy or ethical restrictions.
